# Telaprevir-based triple therapy following partial splenic arterial embolization for chronic hepatitis C with thrombocytopenia can reduce carcinogenesis and improve hepatic function reserve

**DOI:** 10.3892/etm.2015.2674

**Published:** 2015-08-07

**Authors:** TORU ISHIKAWA, SATOSHI ABE, YUICHI KOJIMA, RYOKO HORIGOME, TOMOE SANO, AKITO IWANAGA, KEIICHI SEKI, TERASU HONMA, TOSHIAKI YOSHIDA

**Affiliations:** Department of Gastroenterology and Hepatology, Saiseikai Niigata Daini Hospital, Niigata 950-1104, Japan

**Keywords:** partial splenic arterial embolization, telaprevir-based triple therapy, thrombocytopenia

## Abstract

Thrombocytopenia in patients with chronic hepatitis C negatively impacts interferon (IFN)-based treatment. The aim of this study was to evaluate the efficacy and safety of telaprevir (TVR)-based triple therapy including IFN for patients who have undergone partial splenic arterial embolization (PSE). Ten patients with thrombocytopenia who were infected with hepatitis C virus (HCV) genotype 1b received 12 weeks of TVR in combination with 24 weeks of pegylated interferon (PEG-IFN)α2b and ribavirin following PSE. A sustained virological response (SVR) was seen in 9 of the 10 patients who could be assessed. Early relapse was seen in 1 patient who had the IL-28B minor allele and a null response to pretreatment. The α-fetoprotein levels of the patients decreased from 17.94±7.30 ng/ml prior to PSE to 4.33±2.41 ng/ml at 6 months after triple therapy (P=0.08). Furthermore, serum albumin levels improved significantly from 3.68±0.49 g/dl pre-PSE to 4.13±0.34 g/dl at 12 months after triple therapy (P=0.043). PSE contributed to the treatment success of triple therapy, particularly for patients who were either treatment-naïve, had a history of relapse or the IL28B major allele. This strategy can reduce carcinogenesis and improve hepatic function reserve.

## Introduction

Treatment outcomes for patients with chronic hepatitis C and thrombocytopenia are typically poor due to the difficulty of providing adequate interferon (IFN) therapy (1). Patients with thrombocytopenia may be ineligible for antiviral treatment or, if able to start therapy, may require a dose reduction or even discontinuation due to the hematological adverse effects. Therefore, splenectomy and partial splenic arterial embolization (PSE) are attracting attention for the treatment of thrombocytopenia with the aim of boosting the efficacy of IFN therapy (2,3). However, the indications for these procedures should be examined carefully only after considering the outcomes of IFN therapy.

Triple therapy with pegylated interferon (PEG-IFN), ribavirin (RBV) and telaprevir (TVR) has emerged as a treatment for patients with chronic hepatitis C genotype 1 and a high viral load, and it has been reported to achieve a high sustained virological response (SVR) (4–7). Nevertheless, there are few published studies concerning the outcomes of this triple therapy subsequent to PSE.

The present study was conducted to determine whether PSE is effective for patients given telaprevir-based triple therapy.

## Materials and methods

### 

#### PSE procedure

The present study targeted 10 patients with chronic hepatitis C and thrombocytopenia who had a platelet count of ≤10×10^4^/ml, and who underwent PSE using the Seldinger technique at Saiseikai Niigata Daini Hospital (Niigata, Japan) prior to receiving PEG-IFN/RBV/TVR triple therapy. In this study, thrombocytopenia was defined as a platelet count of <10×10^4^/ml (8). To perform PSE, a hook-shaped 4-Fr catheter (Seiya; Medikit Co., Ltd., Tokyo, Japan) was inserted near to the splenic hilum, and following splenic arteriography, each branch was identified from the intrasplenic artery map that was obtained. A microcatheter (Sniper 2; Terumo Clinical Supply Co. Ltd., Tokyo, Japan) was selectively inserted into each of these vessels, and embolization using coils and 2-mm^2^ gelatin sponges soaked with 20 mg gentamicin (Gentacin; Merck & Co., Inc., Whitehouse Station, NJ, USA) was performed, as previously described (9). To prevent complication, superselective angiography was performed to accurately estimate the areas of infarction and a sterile technique was cautiously practiced during the PSE. Furthermore, broad-spectrum antibiotics were administered by peripheral intravenous infusion for 5 days after the procedure, and oral non-steroidal anti-inflammatory drugs were given as required for fever ≥38°C. The splenic infarction rate of PSE was measured immediately following the procedure by computed tomography angiography.

After confirming an increase in platelet counts, treatment with PEG-IFN/RBV/TVR was initiated, and viral variation and shifts over time in α-fetoprotein (AFP) and serum albumin (Alb) values were investigated.

Mean integration values of Alb and AFP in each patient were calculated prior to and following IFN therapy in order to evaluate the effect on hepatocarcinogenesis of changes in serum Alb and AFP levels during IFN therapy.

Written informed consent was obtained from all patients prior to starting the study, and the Ethics Committee of Saiseikai Niigata Daini Hospital approved the study, which was conducted in accordance with the 1975 Declaration of Helsinki.

#### Antiviral treatment

Each patient received a combination treatment comprising TVR (Telavic; Mitsubishi Tanabe Pharma, Osaka, Japan) at a dose of 750 mg every 8 h in combination with PEG-IFNα2b (PegIntron; MSD, Tokyo, Japan) and RBV (Rebetol; MSD) for 12 weeks, followed by an additional 12 weeks of treatment with only PEG-IFNα2b and RBV. PEG-IFNα2b was injected subcutaneously once weekly at a dose of 1.5 µg/kg. RBV was given orally at a daily dose of 600–800 mg based on body weight. If marked anorexia, an elevation of serum creatinine level or severe anemia developed, the TVR dose was reduced to 1,500 mg/day (750 mg at 12-h intervals after a meal). The method of RBV/TVR dose reduction in the case of anemia was conducted as previously reported (10).

#### Virological assessment and definition of viral response

Serum hepatitis C virus (HCV) RNA level was quantified with the COBAS® TaqMan® HCV test, version 2.0 (detection range 1.2–7.8 log IU/ml; Roche Diagnostics, Branchburg, NJ, USA). The serum HCV RNA level of each patient was assessed prior to treatment, every 4 weeks during treatment and 24 weeks after the therapy.

A rapid virological response was defined as undetectable serum HCV RNA at week 4, and early virologic response as undetectable serum HCV RNA at week 12. SVR was defined as undetectable serum HCV RNA at 24 weeks after the discontinuation of treatment.

#### Statistical analysis

Statistical processing was performed using StatView version 5.0 software (SAS Institute, Cary, NC, USA). All reported P-values are 2-sided, with P<0.05 considered statistically significant. A P-value of <0.05 was considered to indicate a statistically significant difference.

## Results

### 

#### Patient characteristics

The mean age of the patients was 58.67±9.91 years (range, 44–71 years), and the male-female ratio was 8:2. Five patients were pretreatment-naïve, four had relapsed and one was a null responder. Eight patients had the IL-28 major allele, and two patients had the IL-28 minor allele (Table I).

#### Effects of PSE

The mean spleen volume was 329.94±254.78 ml (range, 114.07–1,028.28 ml), the mean infarction volume was 172.55±111.44 ml (range, 31.9–373.3 ml) and the mean splenic infarction rate was 57.32±23.31% (range, 14.8–95.6%; Table I). The mean platelet count increased from 85,600/ml pre-PSE to 152,700/ml post-PSE.

#### Treatment efficacy

SVR was observed in 9 of the 10 patients who could be assessed. Early relapse was seen in 1 patient who had the IL-28B minor allele and a null response to pretreatment (Table II).

**Figure 1.**
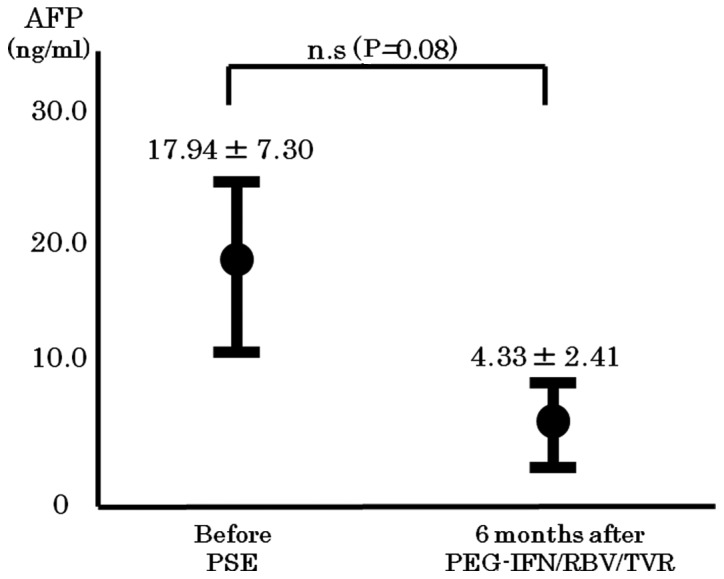
Changes of AFP levels prior to PSE and at 6 months after PEG-IFN/RBV/TVR. AFP, α-fetoprotein; PSE, partial splenic arterial embolization; PEG-IFN, pegylated interferon; RBV, ribavirin; TVR, telaprevir; n.s., not significant.

The mean AFP level of the patients decreased from 17.94±7.30 ng/ml prior to PSE to 4.33±2.41 ng/ml at 6 months after the triple therapy (P=0.08; Fig. 1).

**Figure 2.**
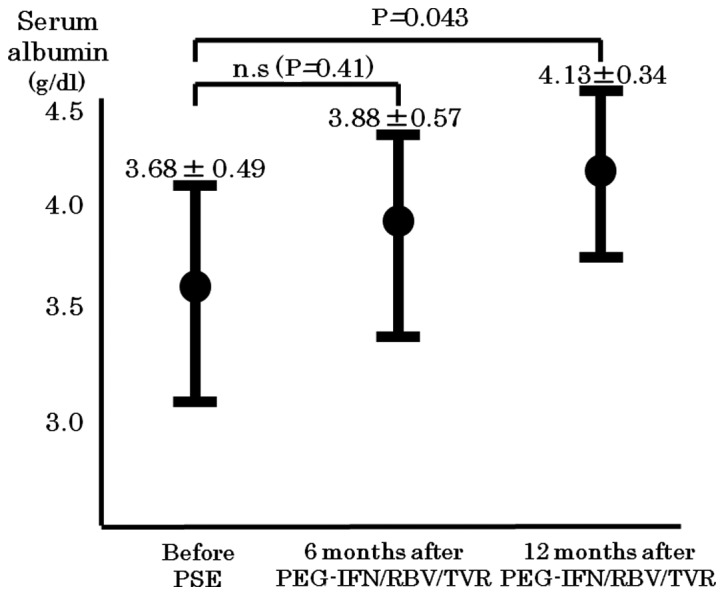
Changes of albumin levels prior to PSE, and at 6 and 12 months after triple therapy. PSE, partial splenic arterial ebolization; PEG-IFN, pegylated interferon; RBV, ribavirin; TVR, telaprevir; n.s., not significant.

The mean serum albumin level of the patients improved from 3.68±0.49 g/dl prior to PSE to 3.88±0.57 g/dl at 6 months after the triple therapy (P=0.41). Furthermore, the mean serum albumin level improved significantly at 12 months after triple therapy from 3.68±0.49 g/dl pre-PSE to 4.13±0.34 g/dl (P=0.043; Fig. 2).

#### Clinical adverse effects

No patients were withdrawn from the study due to adverse reactions to the triple therapy. Four of the 10 patients exhibited thrombocytopenia during the study period, but none had to be withdrawn as a result. A rash occurred in 4 patients, but all cases were Grade 2 or lower.

## Discussion

In patients with advanced hepatitis, thrombocytopenia can be caused by shifts of platelet distribution to an enlarged spleen (11), platelet destruction due to an immunological mechanism (12,13) and decreased production of thrombopoietin (14,15). These patients are at a high risk of hepatocellular carcinoma (HCC). HCC, in particular, is a frequent complication of cirrhosis. IFN therapy is essential in such patients deemed to be at high risk of HCC, and the risk of HCC is expected to decline significantly in those who show a SVR. However, thrombocytopenia represents a major obstacle to IFN therapy. In patients with marked thrombocytopenia with hypersplenism, combination therapy with PEG-IFN or RBV is difficult due to IFN-accelerated thrombocytipenia, and triple therapy is likely to be inadequate.

Splenectomy and PSE have attracted interest for the treatment of portal hypertension with thrombocytopenia (16,17). Splenectomy is considered to be superior to PSE with regard to its ability to increase platelet counts; however, portal vein thrombosis and overwhelming postsplenectomy infection (OPSI) due to pneumococcus are problems (18). OPSI has a high mortality rate and poor prognosis (19,20), and these risks are expected to rise further in patients with progressive liver fibrosis.

Maddison first reported splenic embolization as a treatment for hypersplenectomy in 1973 (21); however, the indications were initially limited by severe complications such as splenic abscess and pneumonia with sepsis. Subsequently, Spigos *et al* reported PSE limited to the area of infarction (22).

As the safety of PSE has improved, the technique has come into wide clinical use. PSE, in a similar manner to splenectomy, increases platelet count, improves hepatic function, hypersplenism, portal hypertension and gastroesophageal varices, and appears effective when used with IFN therapy for type C hepatitis with thrombocytopenia and HCC therapy (23,24). PSE has been demonstrated to be effective for the treatment of thrombocytopenia caused by hypersplenism and for improving liver function (25,26). It is also reportedly effective when combined with IFN therapy for hepatitis C with thrombocytopenia (27); however, the invasive nature of the procedure means that its indications are limited to those in which it is expected to deliver an adequate response.

In Japan, PEG-IFN (plus RBV) therapy is mainly used in patients with Child-Pugh class A cirrhosis subsequent to splenectomy or PSE. The two approaches yield increased platelet counts following treatment in the majority of patients, and treatment outcomes indicate a high SVR rate in those with genotype 2 (28). Therefore, it is considered that a high SVR should be a prerequisite for undertaking invasive procedures such as splenectomy or PSE.

Even patients with hepatitis C genotype 1 and a high viral load who are refractory to dual therapy with PEG-IFN/RBV have exhibited a high SVR when receiving triple therapy with PEG-IFN/RBV/TVR, giving rise to expectations of expanded indications for PSE. In the present study, triple therapy consisting of PEG-IFN/RBV/TVR was used following PSE in 10 patients, including a patient with chronic hepatitis C genotype 1, a high viral load and thrombocytopenia, with the aim of achieving a high SVR. By opting to perform PSE in patients with chronic hepatitis C and thrombocytopenia, it was possible to initiate triple therapy and achieve a high SVR. Since this treatment modality has been found to reduce AFP values, it may help to inhibit carcinogenesis in patients with elevated AFP values who are at high risk of cancer due to liver fibrosis. Furthermore, the administration of PEG-IFN/RBV/TVR following PSE increased albumin levels and improved hepatic functional reserve through the antiviral effects. These benefits demonstrated by the combination therapy suggest that is may contribute to an improved prognosis for patients with chronic hepatitis C and thrombocytopenia.

It is recommended that a high SVR is a requirement when attempting invasive procedures such as PSE. Nevertheless, 1 patient with the IL-28B minor allele and a null response to pretreatment suffered an early relapse. This finding indicates that patients undergoing triple therapy in combination with PSE should either have the IL-28B major allele or a previous treatment history of relapse. With the exception of refractory patients, we consider that many patients should be eligible for this form of treatment, provided that sufficient care is taken to prevent PSE-associated complications.

Based on these data, it is possible to make more effective decisions concerning antiviral treatment options for patients and whether PSE should be performed. It is also important to monitor patients undergoing antiviral treatment for the development of HCC.

We consider that increased platelet counts following PSE will facilitate the safe use of PEG-IFN/RBV/TVR triple therapy in patients with chronic hepatitis C and thrombocytopenia. A further study with a larger number of patients is required to confirm the findings of the present study.

In conclusion, triple therapy combined after PSE is indicated to be an acceptable treatment that is expected to inhibit carcinogenesis in patients with chronic hepatitis C and thrombocytopenia perceived to be at high risk of cancer due to fibrosis of the liver.

## Figures and Tables

**Table I.** Clinical features of patients with thrombocytopenia and chronic hepatitis C.

Case no.	Age (years)	Gender	Treatment history	IL-28	Spleen volume (ml)	Infarction volume (ml)	Non-infarction volume (ml)	Infarction rate (%)
1	49	F	Naïve	Major	268.6	131.3	137.3	48.9
2	69	M	Relapse	Major	217.1	167.4	49.7	77.1
3	44	M	Relapse	Major	1,028.2	364.8	663.4	35.4
4	56	F	Relapse	Minor	117.4	112.4	5.0	95.6
5	67	M	Naïve	Major	114.1	79.6	34.5	69.2
6	71	M	Relapse	Major	215.6	31.9	183.7	14.8
7	71	M	Naïve	Major	210.9	106.6	104.3	50.6
8	46	M	Naïve	Major	338.2	248.4	89.8	73.3
9	55	M	Naïve	Major	283.4	117.8	165.6	41.6
10	61	M	Null	Minor	482.2	373.3	108.9	46.5

M, male; F, female.

**Table II.** Virological response of telaprevir-based triple therapy after partial splenic arterial embolization.

	HCV RNA (Log IU/ml)				
		
Case no.	Pre treatment	4 weeks	12 weeks	EOT	SVR
1	7.3	–	–	–	Yes
2	7.1	–	–	–	Yes
3	6.6	–	–	–	Yes
4	5.6	–	–	–	Yes
5	6.2	–	–	–	Yes
6	7.3	–	–	–	Yes
7	6.8	–	–	–	Yes
8	6.2	–	–	–	Yes
9	5.4	–	–	–	Yes
10	5.6	–	–	–	Relapse^a^

a Relapse at 4 weeks. Antiviral response after 4, 12, and 24 weeks (end of treatment, EOT). A dash (−) indicates undetectable levels. HCV, hepatitis C virus; SVR, sustained virological response.
